# Inkjet printing of mechanochromic fluorenylidene-acridane

**DOI:** 10.1038/s41598-022-21600-x

**Published:** 2022-10-10

**Authors:** Keisuke Ogumi, Kohki Nagata, Yuki Takimoto, Kentaro Mishiba, Yutaka Matsuo

**Affiliations:** 1 Department of Chemical Systems Engineering, Graduate School of Engineering, Nagoya University, Furo-cho, Chikusa-ku, Nagoya, 464-8603 Japan; 2 Tokyo Metropolitan Industrial Technology Research Institute, 2-4-10 Aomi, Koto-ku, Tokyo, 135-0064 Japan; 3 Institute of Materials Innovation, Institutes of Innovation for Future Society, Nagoya University, Furo-cho, Chikusa-ku, Nagoya, 464-8603 Japan; 4 Department of Mechanical Engineering, School of Engineering, The University of Tokyo, 7-3-1 Hongo, Bunkyo-ku, Tokyo, 113-8656 Japan

**Keywords:** Mechanical properties, Optical materials

## Abstract

In mechanochromic material research, a serious problem is that mechanical treatment cannot be applied to the materials because of their responsiveness to stimuli. Inkjet printing is a useful solution deposition method for electronics, but materials must be processed to be suitable for an inkjet printer. Fluorenylidene-acridane (FA) exhibits ground-state mechanochromism with visual color changes and responds not only to mechanical pressure but also to alcohol. Alcohol inhibits the color change induced by mechanical stimulation because the mechanochromism of FA is based on a conformational change in its molecular structure. This phenomenon suggests that the mechanochromism of FA can be controlled using alcohol. For use in inkjet printing, minute particles of FA obtained by bead milling in ethanol were investigated for uniformity and size by scanning electron microscopy and gas adsorption measurement. Also, ink containing FA particles was prepared and examined for physical properties such as viscosity and surface tension. It was confirmed that the inkjet-printed pattern demonstrated visual color changes between yellow and green in response to mechanical pressure and alcohol. This report describing the control of mechanochromism and its specific application is expected to contribute to broadening the mechanochromic materials research field.

## Introduction

Mechanochromic materials are expected to be applied in pressure sensing, display devices, and recording media owing their responsiveness to mechanical pressure^1–4^. For those applications, thin films must be prepared by vacuum deposition or solution fabrication processes such as spin coating, deposition coating, and bar coating. Although solution-based coating methods are advantageous for printed electronics, the above methods waste much of the solution containing a dissolved material. Inkjet printing technology offers a way to solve this problem, because it can print desired patterns without wasteful loss of material. Owing to its simplicity, low cost, and ease of application, inkjet printing has been studied in various fields, printable devices^5–7^, including transistors^8^, OLEDs^9^, supercapacitors^10^, and biomedical engineering^11^. Inkjet printing requires micronization and homogenization of the material to be printed. Given that the properties of mechanochromic materials are altered by mechanical stimuli, these treatments are critical problems hindering the application of the materials not only in inkjet printing but also in other areas. For this reason, while many reports have discussed the mechanisms, behavior and potential benefits of mechanochromic materials^12–19^, there are few examples of specific applications^20–23^.

**Figure 1:**
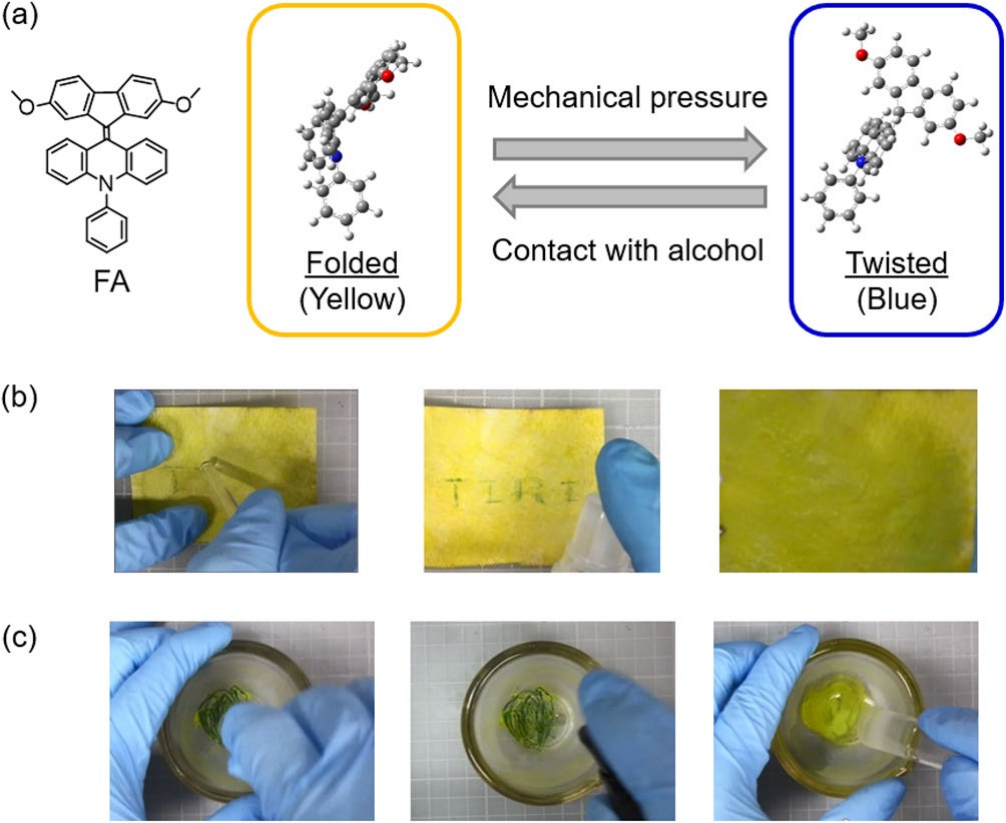
(**a**) Molecular structure of FA and ground-state mechanochromism. (**b**) Resetting the mechanochromism using ethanol. (**c**) Prevention of mechanochromism in the presence of ethanol.

In the past few years, we have researched fluorenylidene-acridane derivatives (FAs) which are examples of overcrowded ethylene derivatives^24–30^ and we reported that FAs show mechanochromic behavior based on a molecular structural change from a folded conformer to a twisted conformer by mechanical pressure (Fig. 1a)^31–33^. While most mechanochromic materials undergo an emission color change, mechanochromism of FAs brings about a unique visual color change. This phenomenon is explained by the photophysical changes of FAs derived from ground-state mechanochromism. Further, in addition to the above mechanical pressure sensing, we found that FAs exhibit a response to alcohol^34^. An FA that has dimethoxy substituents on the fluorene unit could be returned from the blue color caused by mechanical pressure to its original yellow color by use of alcohol (Fig. 1b). Also, FA in alcohol keeps its yellow color even when it is ground with a mortar and pestle (Fig. 1c). This observation means that the mechanochromism of FA could be controlled in the presence of alcohol.

In this study, we performed bead milling of FA in ethanol to obtain uniform minute particles without mechanochromic behavior and then those particles were used for inkjet printing to demonstrate a manufacturing application. Herein, we controlled the particle size of FA and investigated the physical properties of the formulated ink such as viscosity, surface tension, and contact angle. The inkjet-printed patterns exhibited two distinct responses to mechanical pressure and alcohol. Because it is well established and widely used, inkjet printing technology could accelerate the development of manufacturing in the mechanochromic materials research field.

## Results and discussion

**Figure 2:**
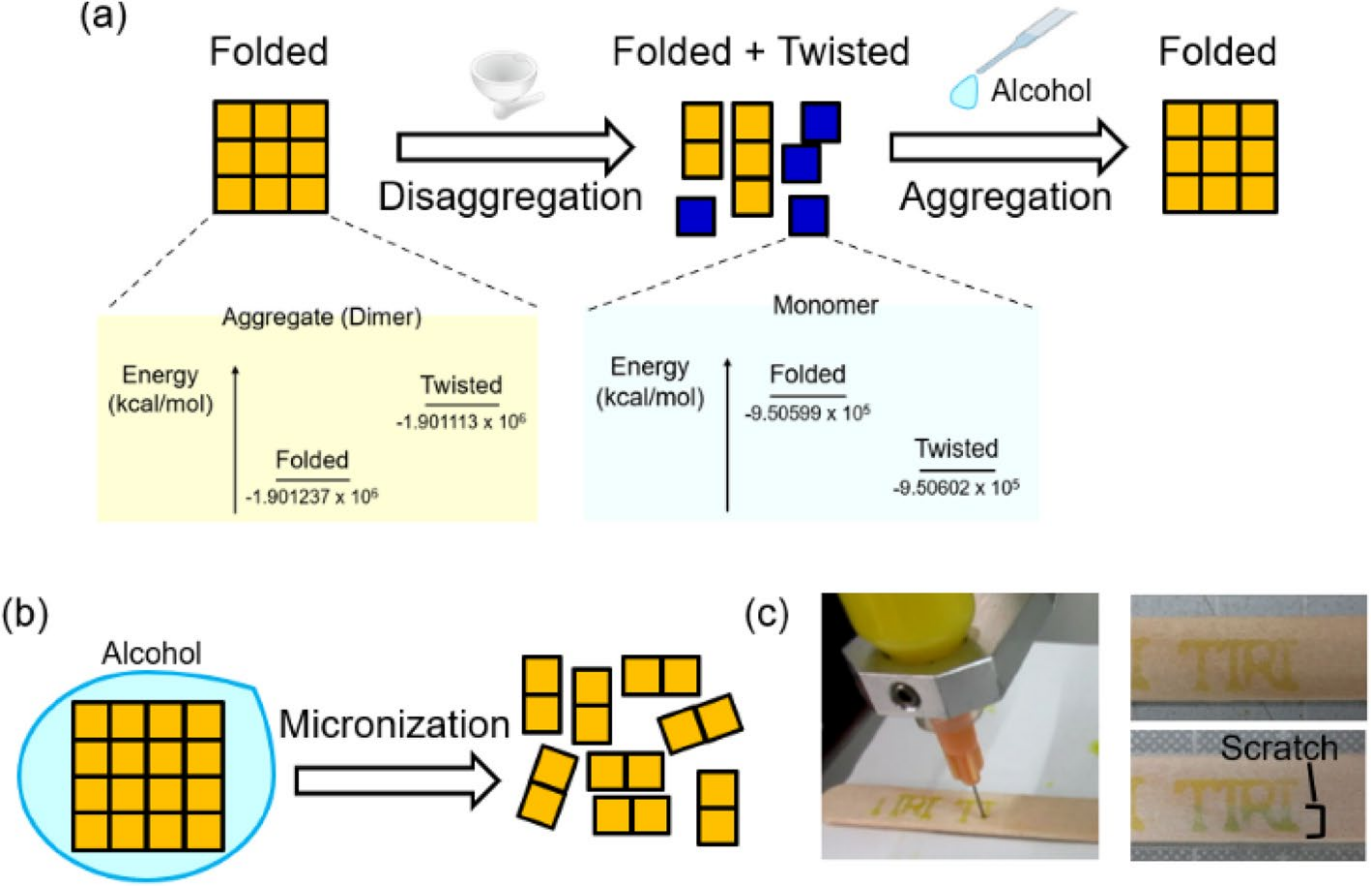
(**a**) Illustration of the relationship among conformation, color change, and aggregation/disaggregation of FA, and calculated energy levels of the conformers (the aggregates were simplified to dimers to reduce the computational cost). (**b**) Micronization while using alcohol to suppress mechanochromism. (**c**) Preliminary demonstration using a dispenser to deposit ground particles of FA.

First, we describe the mechanochromism of FA and its response to alcohol. A visual color change occurs due to a change in molecular structure from a folded conformer to a twisted conformer. Theoretical calculations showed that the conformational change is caused by aggregation and disaggregation (Fig. 2a). While the folded conformer is stable in the aggregated state because of the intermolecular interaction energy, the twisted conformer is preferred in the disaggregated stated based on a comparison of stabilization energies. Therefore, disaggregation by mechanical pressure causes the conformational change from the folded to twisted structure, inducing a color change from yellow to green. Subsequently, upon contact with alcohol, which is a poor solvent for FA, the twisted conformers aggregated and returned to the folded conformation in accordance with its stabilized energy levels. Because alcohol promoted aggregation, the fine particles of FA remained in the folded conformation even when ground with a mortar and pestle. Control of the mechanochromism using alcohol enabled the preparation of uniform minute particles of FA without causing a color change. (Fig. 2b). Also, realizing that the hydroxyl group of alcohol promoted both aggregation and retention of the yellow color, we confirmed that polyvinyl alcohol (PVA) with excess hydroxyl groups produced a response similar to alcohol. PVA is a useful polymer owing to its water solubility, low environmental impact, and safety in terms of human health, so there have been various reports on the use of PVA in electronics^35–39^ and medical research^40,41^. Thus, in the present work, we employed PVA as a dispersant, thickener, and binder for inkjet printing.

In a preliminary demonstration before inkjet printing, we produced ink consisting of aqueous PVA solution and FA ground with a mortar and pestle. Then, the ink was loaded into a dispenser. While pristine FA is not suitable for dispensing, yellow characters would be written on a wooden board when using the pulverized FA in aqueous PVA solution. After the solvent had evaporated, the printed characters exhibited visual mechanochromism from yellow to green (Fig. 2c). This preliminary demonstration showed the feasibility of performing mechanical treatment of FA without inducing a mechanochromic response.

**Figure 3:**
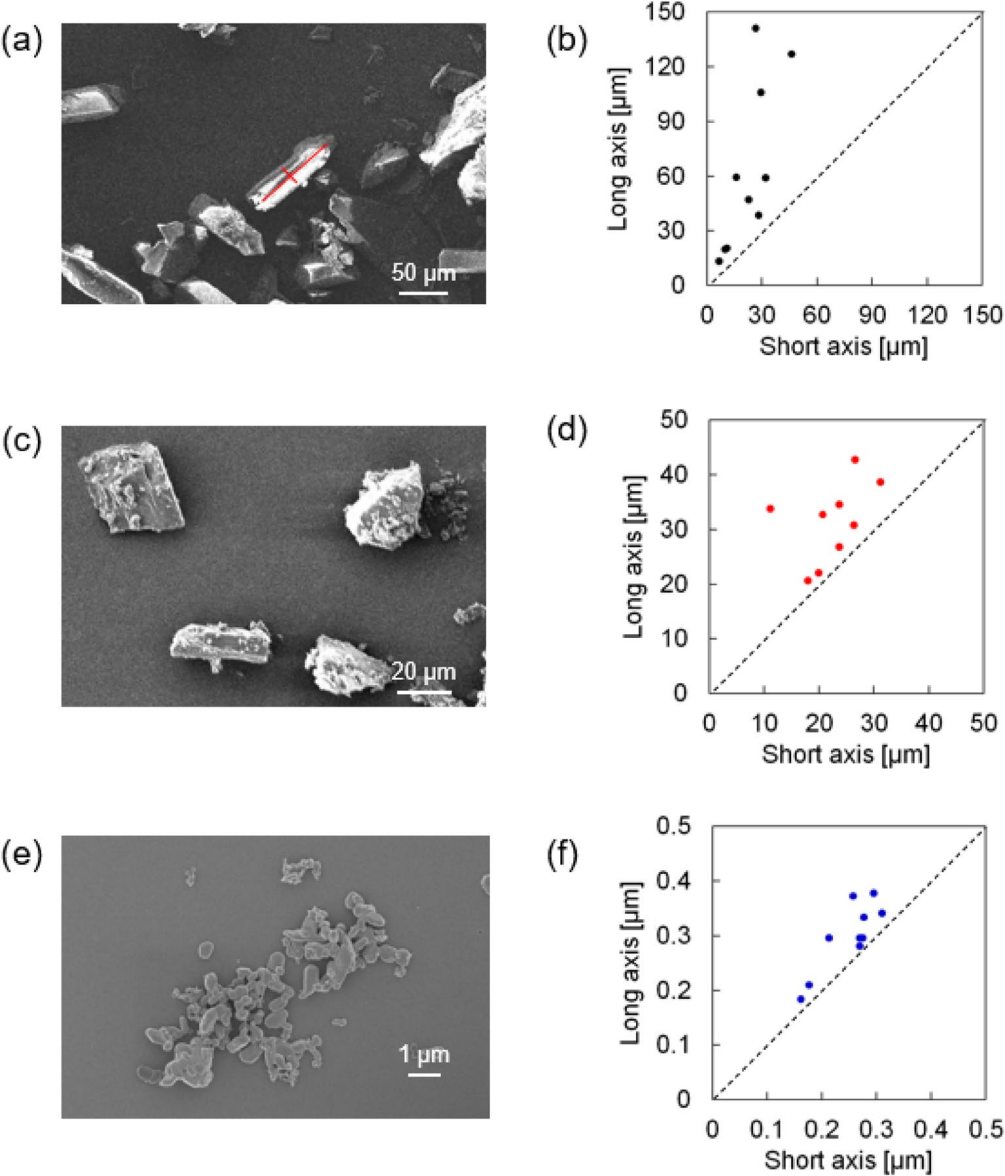
SEM images of (**a**) pristine, (**c**) ground, and (**e**) bead-milled FA. Graphs of the long axis versus the short axis of 10 particles for (**b**) pristine, (**d**) ground, and (**f**) bead-milled FA.

**Figure 4:**
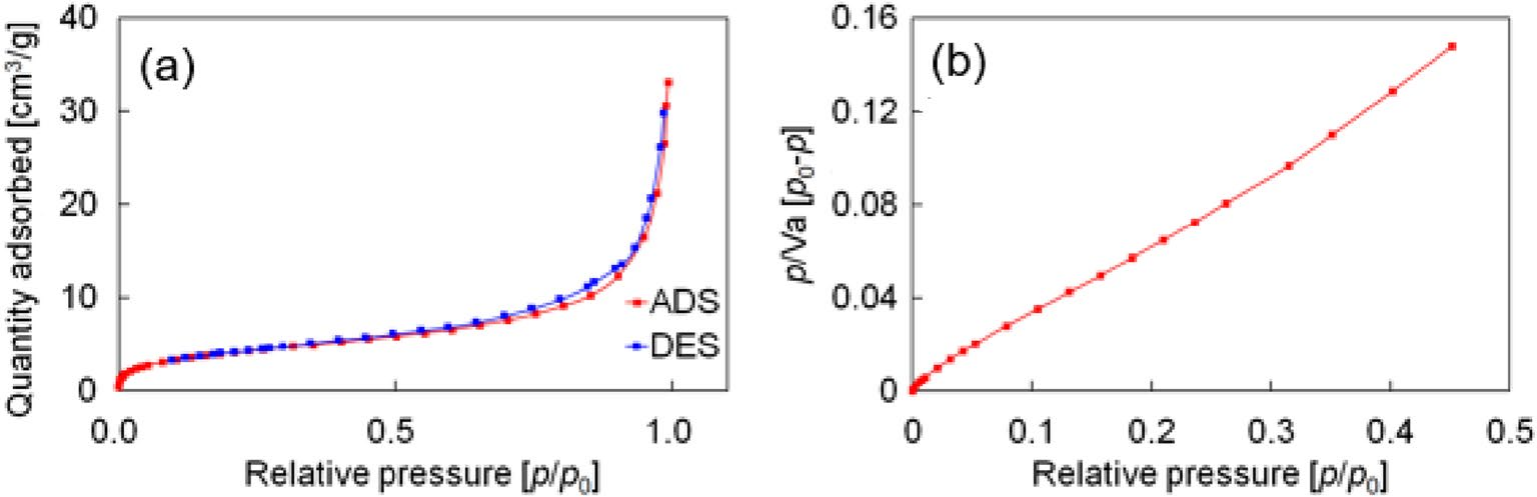
Gas adsorption measurement of bead-milled FA. (**a**) Adsorption isotherm. (**b**) BET plot.

Drop-on-demand inkjet printing is roughly classified into thermal systems and piezoelectric systems based on the method of discharging ink from a printhead. In these respective systems, ink is discharged due to a pressure pulse generated by vapor bubbles under heating or by deformation of a piezoelectric element. Considering the thermal stability of FA and the fact that thermal systems increase the ink temperature to approximately 300 °C, we opted for a piezoelectric inkjet printer. To use FA in inkjet printing, we worked to control its particle size. Although the diameter of nozzle of printhead employed in this study was 80 μm, we aimed the particles of less than 1 μm in diameter because this value is required for standard inkjet printer. Thus, the particle size of FA was investigated for the pristine powder, powder ground with a mortar and pestle, and powder processed with a bead mill. Bead milling was performed using ZrO_2_ beads of 0.05 mm in diameter in ethanol at 2000 rpm for 1 h. Scanning electron microscopy (SEM) revealed that the pristine particles were fine crystals with a rod-like shape (Fig. 3a). In mechanical treatment conditions using a mortar (Fig. 3c) or bead milling (Fig. 3e), the particles became a roundish shape. The graphs plotted in Fig. 3b, d, and f show the correlations between the long axis and short axis of particles prepared under the three conditions (pristine, ground, and bead-milled). It can be seen that the difference between the long axis and short axis became smaller in the order of pristine, ground, and bead-milled. The average size of 10 particles and the standard deviations under each condition are summarized in Table 1. Although the average sizes of pristine and ground FA were over 1 μm, bead-milled FA was found to be suitable for the inkjet printer because it had a particle size of 200–300 nm and small standard deviation. Gas adsorption measurements were consistent with the trend in particle size observed by SEM (Figs. 4, S2, and S3, and Table 2). Compared with the pristine and ground powder, the bead-milled powder had a much higher BET surface area. Therefore, we concluded the bead milling treatment was necessary for use of FA in inkjet printing. The bead-milled powder demonstrated the same mechanochromic response compared with the pristine (Movie S1 and S2).

**Table 1:** Average size and standard deviation of 10 particles prepared under each condition.

		Size ave. [μm] (N = 10)	Standard deviation
Pristine	Short side	23.05	11.52
	Long side	63.03	43.64
Ground	Short side	24.77	8.82
	Long side	34.34	10.96
Bead-milled	Short side	0.251	0.047
	Long side	0.298	0.060

**Table 2:** BET surface area from gas adsorption measurement.

	Pristine	Ground	Bead-milled
BET surface area [m^2^/g]	< 0.1	0.62	15.1

**Figure 5:**
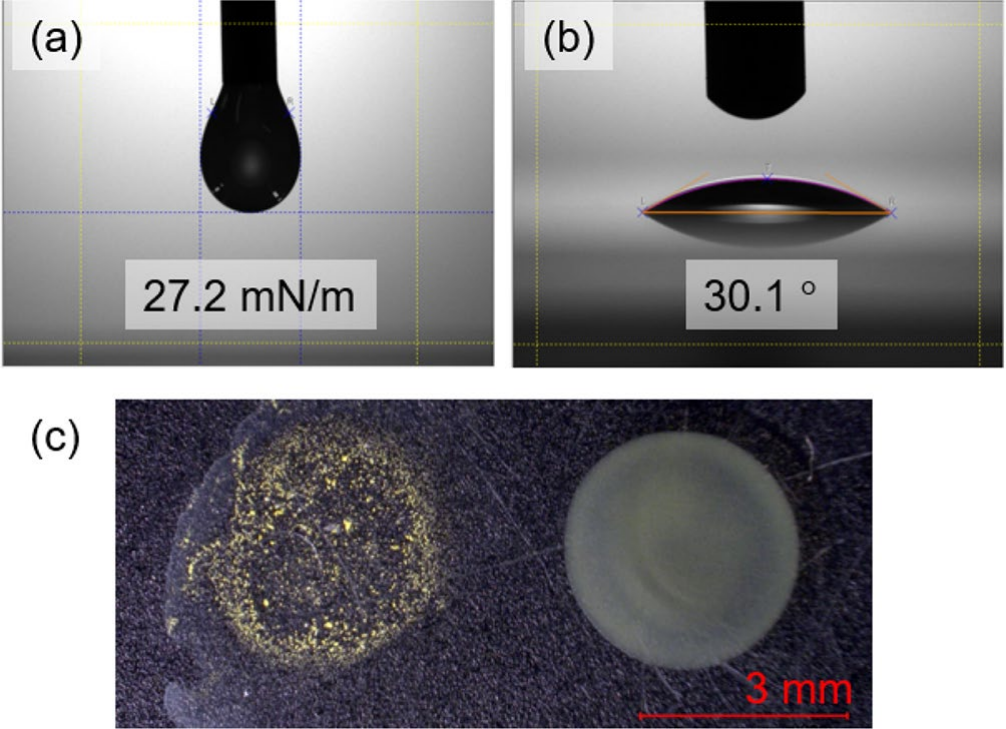
Photographs of (**a**) surface tension and (**b**) contact angle measurements. (**c**) Tests for the coffee ring effect (left: only FA in ethanol; right: formulated ink).

Next, we formulated the ink using FA, ethanol, PVA, and pure water. Generally, ink viscosity of less than 20 cP (mPa/s) is required for a piezoelectric inkjet printer^42^. We adjusted the viscosity by adding saturated aqueous PVA solution to ethanol. According to measurements with a rheometer, a 10:1 volume ratio of ethanol to saturated aqueous PVA achieved the required viscosity (Figure S4). This ratio was also sufficient to disperse the bead-milled FA particles, whereas the particles precipitated when the ratio was 20:1 (Fig. S5). Surface tension of ink is an important factor that affects the discharge rate from a printhead nozzle, with a range of 25–75 mN/m needed for an inkjet printer^43^. For the formulated ink, we estimated measured the surface tension to be 27.2 mN/m and the contact angle to be 30.1° by the pendant drop method (Fig. 5a and b). These values of the formulated ink are suitable for inkjet printing. Surface tension also affects the uniformity of printed patterns. When the solvent of the printed pattern evaporates, the solute is generally deposited at the edge of the pattern. This phenomenon is the well-known coffee ring effect, which is derived from Marangoni flow based on the difference in surface tension between the edge and the interior^44^. Various methods for preventing the coffee ring effect have been reported, such as using mixed solvents^45^, using additives^46,47^, and controlling the substrate temperature^48^. When we dropped the formulated ink on a glass substrate, FA was dispersed all throughout the droplet. As shown Fig. 5c, the coffee ring effect was not observed with the formulated ink, but was observed in the case without PVA. This observation suggests that the viscosity imparted by PVA was useful for inhibiting Marangoni flow.

**Figure 6:**
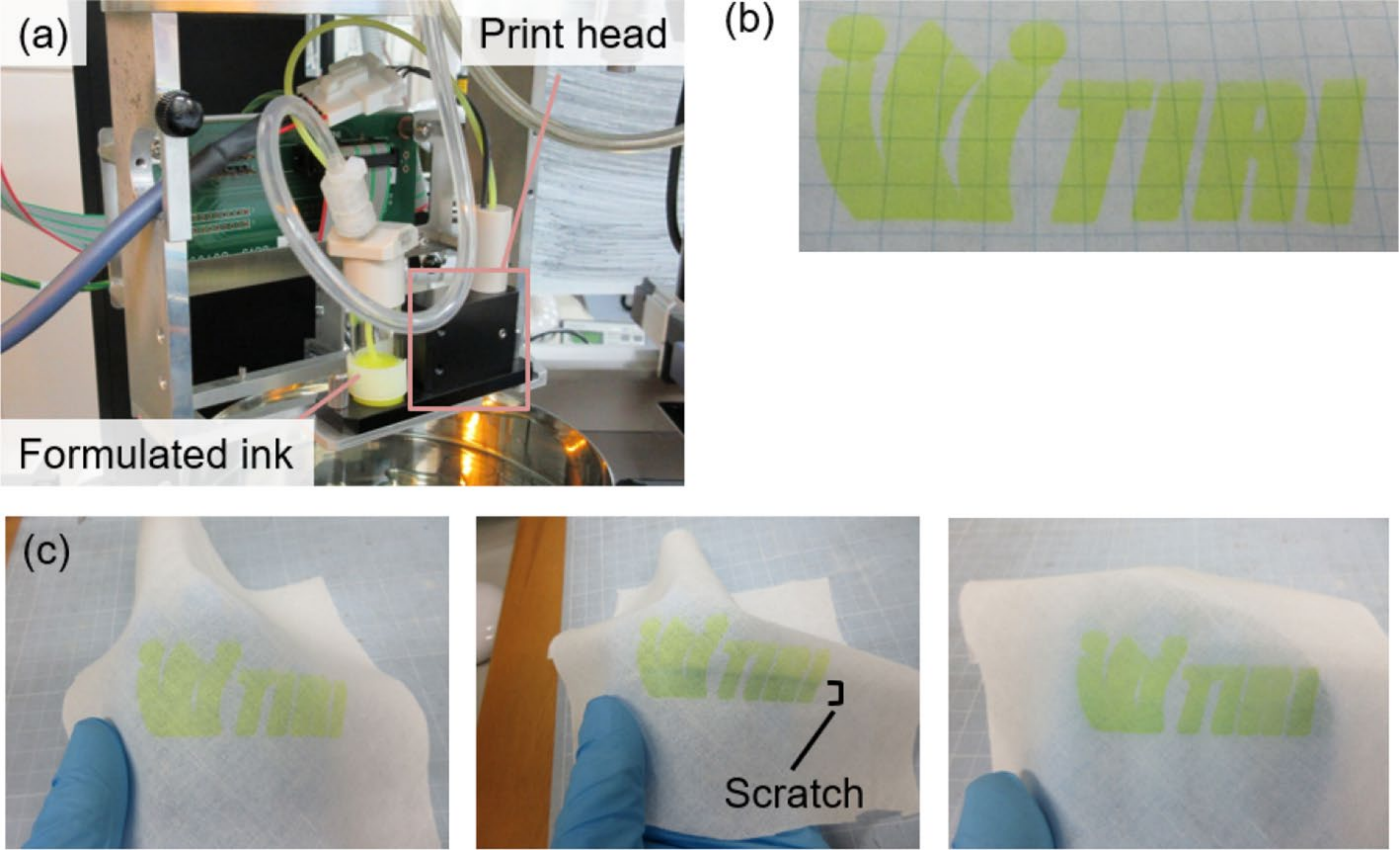
(**a**) Photograph of application in an inkjet printer. (**b**) Printed pattern. (**c**) Printed pattern on a fabric substrate. Response to mechanical pressure and ethanol.

Subsequently, we used the formulated ink for inkjet printing (Fig. 6a). Detailed printing parameters are shown in Table S1. Simple patterns such as straight lines and circles could be well drawn as shown in Fig. S6a. Next, more complex patterns were printed on paper (Fig. 6b). This pattern exhibited mechanochromic behavior in response to mechanical pressure, the same as pure FA (Figure S6c). Further, the inkjet printer could draw patterns on a fabric substrate. This printed fabric sample responded to mechanical pressure and alcohol with visual color changes (Fig. 6c and Movie S3). Interestingly, even after PVA was removed by stirring in water, the printed pattern remained intact (Fig. S7a and b). This result shows that the fabric sample was a washable, flexible, and repeatable mechanochromic material.

## Conclusion

Responsiveness to mechanical stimuli has inhibited the development and practical application of mechanochromic materials. This crucial problem is the reason why few studies have demonstrated specific applications of mechanochromic materials. We focused on the response of FA to alcohol and used this property as a means to control the mechanochromic behavior of FA. This idea enabled preparation of minute particles of FA by micronization without causing mechanochromism. To demonstrate the application of mechanochromic materials, we applied the minute particles of FA in inkjet printing. The size and uniformity of FA particles prepared by bead milling were determined by SEM. A formulated ink using FA, ethanol, and saturated aq. PVA was investigated in terms of physical properties such as viscosity, surface tension, and contact angle. Then, this ink was used in a piezoelectric inkjet printer to draw a pattern that exhibited reversible color changes in response to mechanical stimuli and alcohol. We expect that the application of FA in inkjet printing technology will be a key point in the development of the mechanochromic materials research field.

## Method

Synthetic procedure of FA was described in Supporting information. Pre-demonstration was performed using a dispenser, SuperΣ CM II/SHOT mini 200Ω (MUSASHI engineering inc.). FA was micronized by Bead milling treatment, Ashizawa Finetech Ltd. HFM02. In this treatment, FA in ethanol was stirred for 1 h at 2000 rpm using ZrO_2_.The particles of FA were observed by SEM, JEOL JSM-6610LA and Hitachi High-Tech Regulus8230. BET surface area was measured by gas absorption equipment, MicrotracBEL BELSORP-max. Viscosity was measured by rheometer, Spectris Kinexus pro^+^. Surface tension and contact angle were investigated by Kyowa Interface Science Co., Ltd. DMo-602. The Coffee ring effect was observed by digital microscope, KEYENCE VHX-1000. Inkjet printing was performed by MICROJET IJHE-1000. Detail printing parameters were described in Table S1.

## Supplementary Information

Supplementary Information 1.

Supplementary Video 1.

Supplementary Video 2.

Supplementary Video 3.

## Data Availability

The datasets used and/or analysed during the current study available from the corresponding author on reasonable request.
