# Supplementary material for: Inkjet printing of mechanochromic fluorenylidene-acridane
Source: Sci Rep. 2022 Oct 10;12:16997. doi: 10.1038/s41598-022-21600-x (PMC9550800; doi:10.1038/s41598-022-21600-x)
Supplement: Supplementary file 1 — Supplementary Information 1. [file 41598_2022_21600_MOESM1_ESM.pdf]

Supporting Information

Inkjet Printing of Mechanochromic Fluorenylidene-Acridane

Keisuke Ogumi[1,2], Kohki Nagata[2], Yuki Takimoto[2], Kentaro Mishiba[2] and Yutaka Matsuo[1,3,4]

[1] *Department of Chemical Systems Engineering, Graduate School of Engineering, Nagoya University, Furo-cho, Chikusa-ku, Nagoya 464-8603, Japan*

[2] *Tokyo Metropolitan Industrial Technology Research Institute, 2-4-10 Aomi, Koto-ku, Tokyo 135-0064, Japan*

[3] *Institute of Materials Innovation, Institutes of Innovation for Future Society, Nagoya University, Furo-cho, Chikusa-ku, Nagoya 464-8603, Japan*

[4] *Department of Mechanical Engineering, School of Engineering, The University of Tokyo, 7-3-1 Hongo, Bunkyo-ku, Tokyo 113-8656, Japan*

Table of Contents:

1. Experimental Section ········ S2

2. Gas adsorption measurement ········ S3-S4

3. Investigation for viscosity of the formulated ink ········ S5

4. Observation for dispersion of FA ········ S5

5. Detail printing parameters ········ S5

6. Application for inkjet printer ········ S6

1. Experimental Section

Materials were purchased from Tokyo Kasei Industry Co., Ltd., Sigma-Aldrich Inc., and other commercial suppliers and used after appropriate purification before use. Anhydrous solvents (stabilizer-free) were purchased from FUJIFILM Wako Pure Chemical Corporation. All reactions dealing with air- or moisture-sensitive compounds were carried out in a dry reaction vessel under nitrogen or argon. All reactions were monitored by thin layer chromatography (TLC, eluent, $CHCl_3$). FA was obtained by Barton-Kellogg reaction using diazofluorenylidene and thioacridane (Figure S1). Diazofluorenylidene (1.90 g, 5.89 mmol) and triphenylphosphine (3.71 g, 14.1 mmol) was added in two-necked flask. Thioacridane dissolved in *o*-xylene was dropped in the flask at 140 °C for 1 hour. Reaction temperature was decreased to room temperature and the solvent was removed by evaporator. The solid products were charged on a silica gel short column to remove some impurities using dichloromethane as eluent. Then, triethylamine/dichloromethane mixture (1/5) was passed to collect the crude. Second silica gel column which was prepared with trimethylamine was performed using *n*-hexane/dichloromethane (1/5) as eluent to obtain the pure twisted conformer of FA**.** The folded conformer of FA (1.19g, 2.47 mmol) was generated from the twisted conformer in methanol by sonication. The product was verified by NMR spectra (JEOL ECA-600).

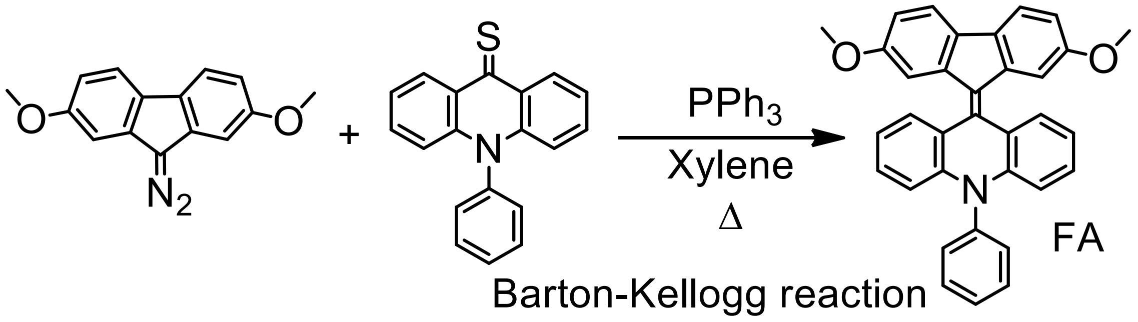

Figure S1. Synthesis of mechanochromic fluorenylidene-acridane FA.

2. Gas adsorption measurement

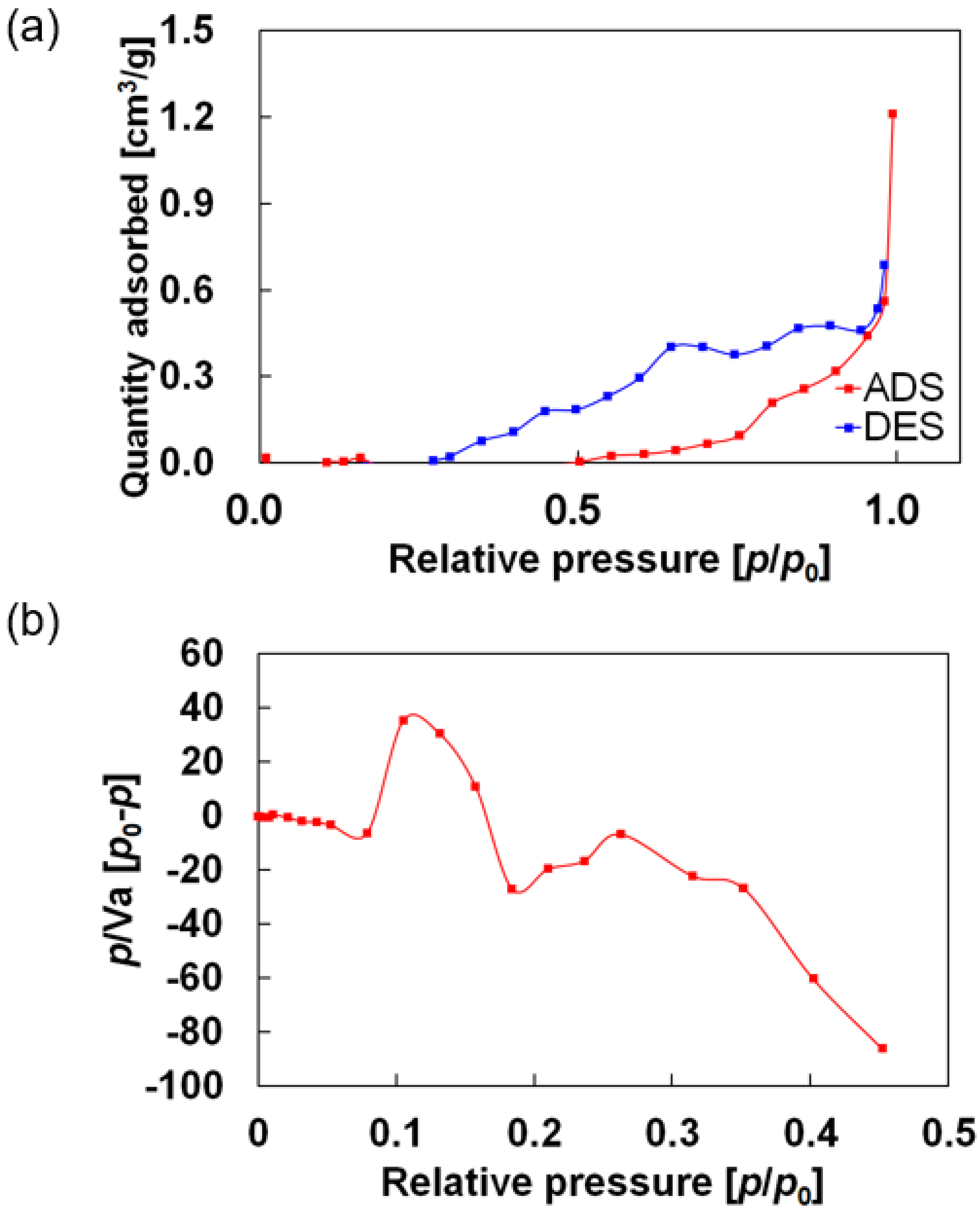

Figure S2. Gas adsorption measurement in the pristine particle (a) Adsorption isotherm. (b) BET plotting graph.

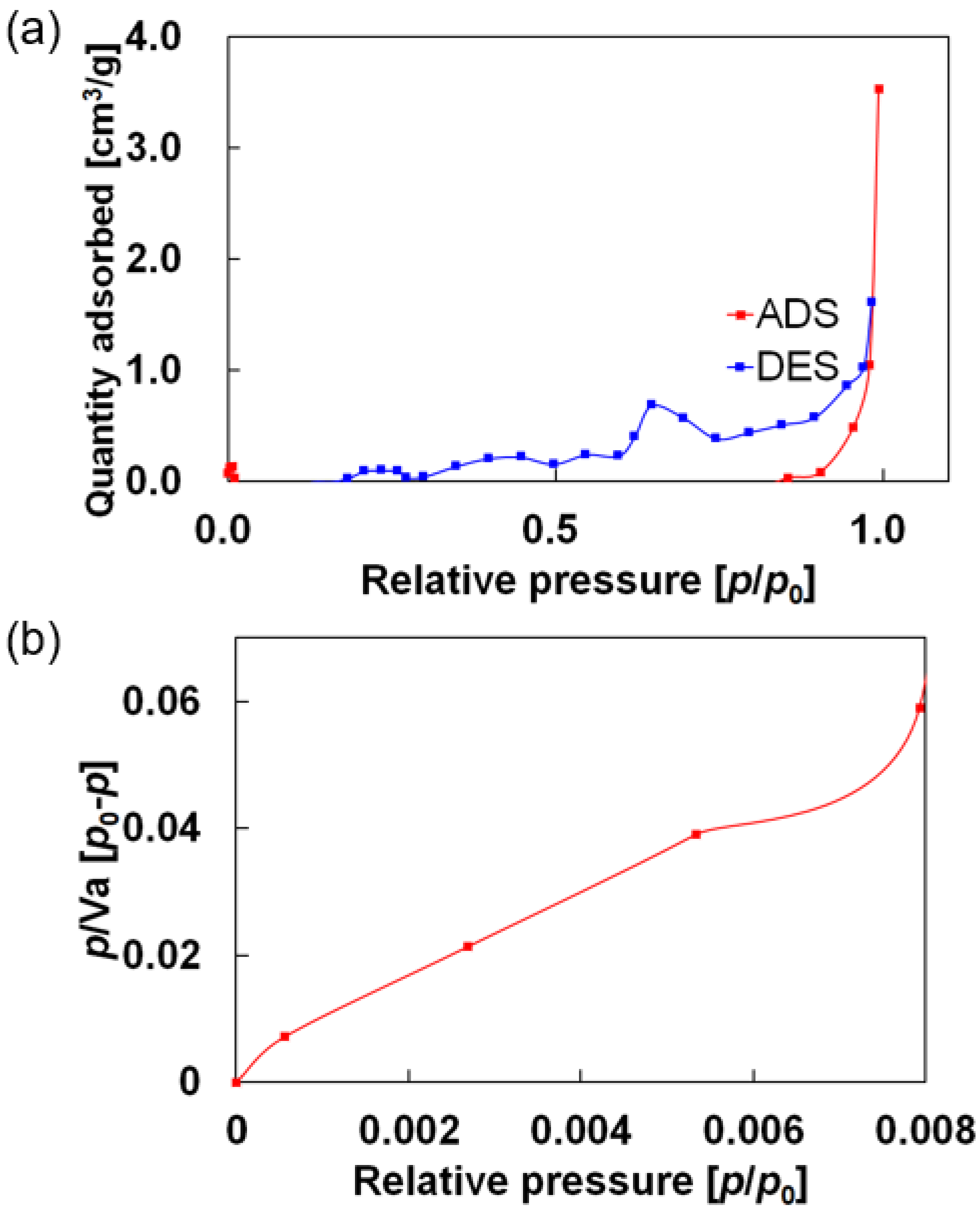

Figure S3. Gas adsorption measurement in the ground particle (a) Adsorption isotherm. (b) BET plotting graph.

3. investigation for viscosity of the formulated ink

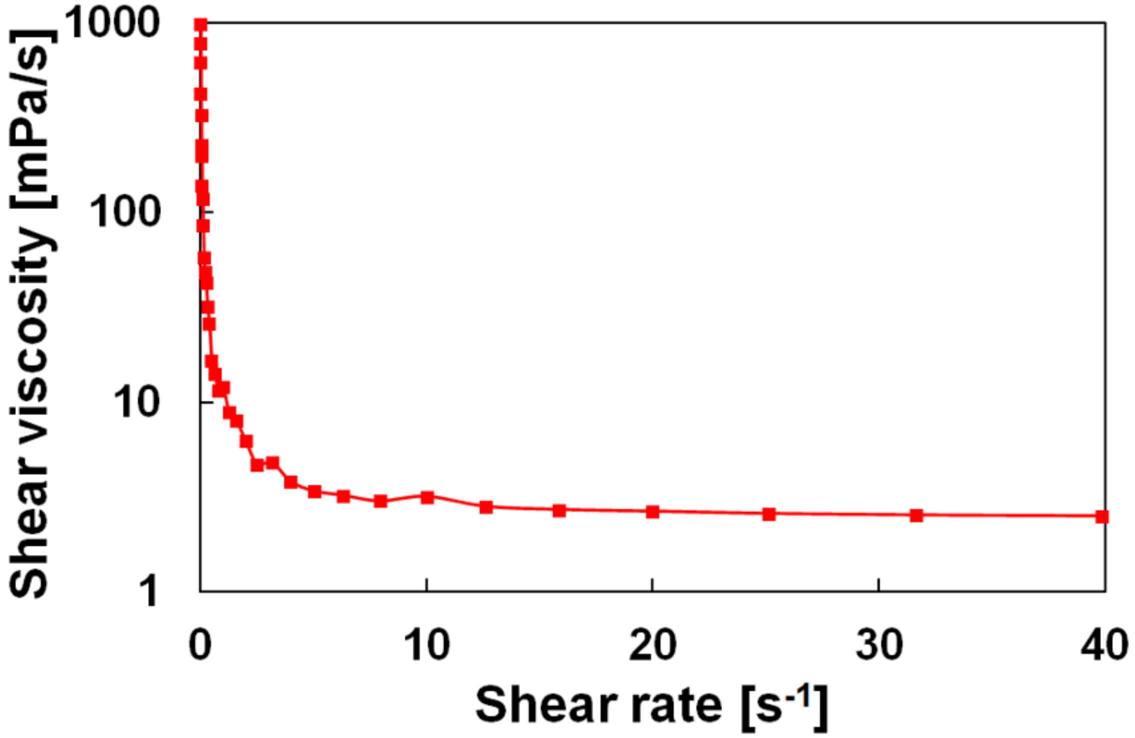

Figure S4. Viscosity of the formulated ink versus shear rate.

4. Observation for dispersion of FA

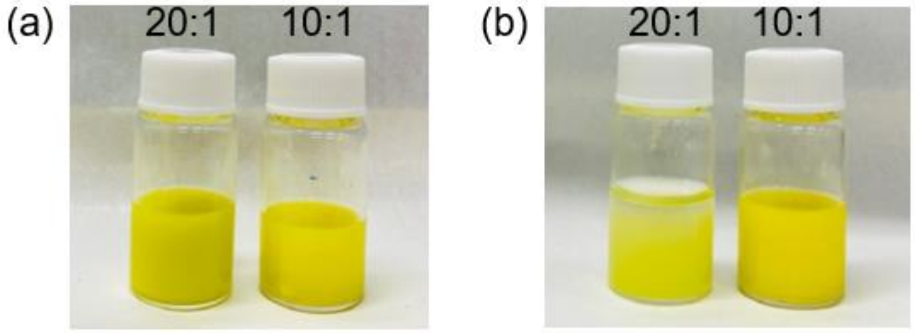

Figure S5. Photographs of the formulated inks (left: ethanol : saturated PVA aq = 20 : 1, right: 10 : 1) (a) Just after formulation. (b) After 3 days.

5. Detail printing parameters

Table S1. Printing parameters of the piezoelectric printhead.

Pulse voltage [V]	Pulse width [μs]	Hertz
80	100	500

6. Application for inkjet printer

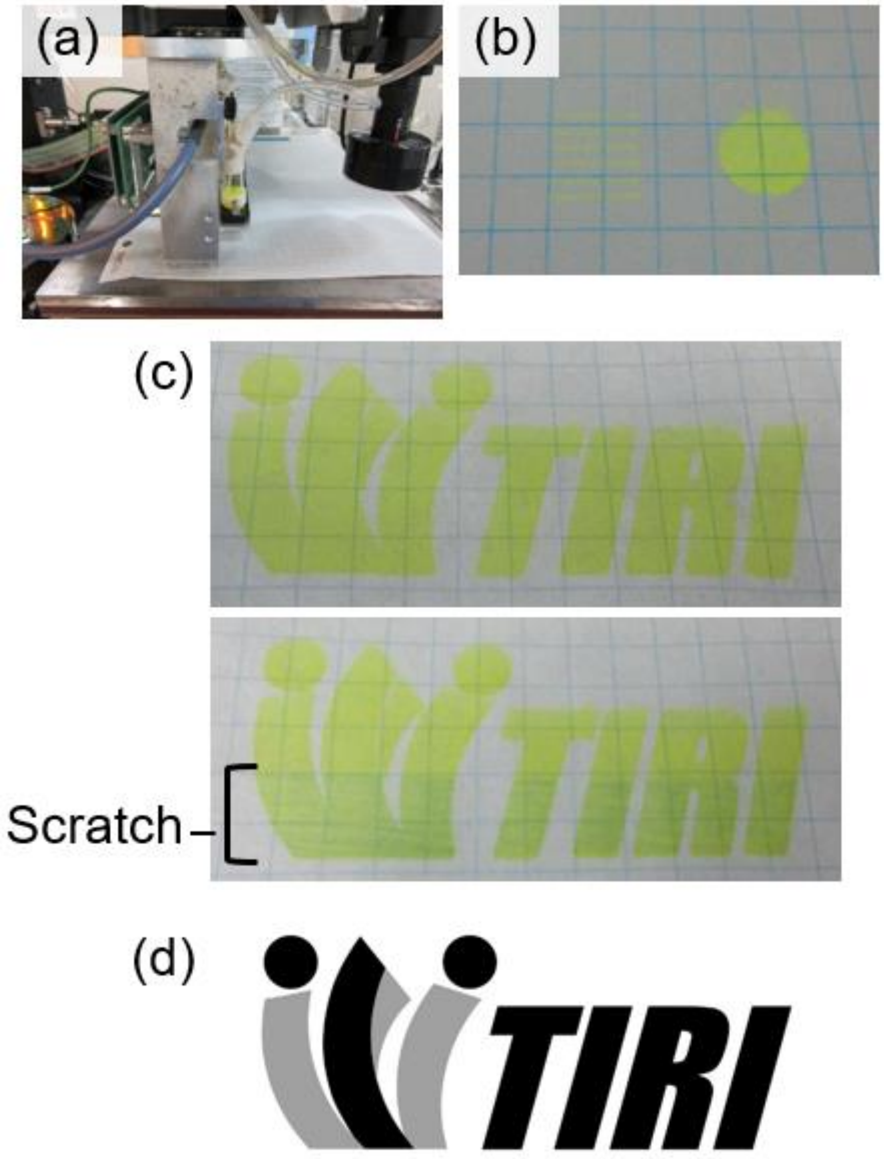

Figure S6. (a) Photograph of printing on a paper. (b) Printed patterns of straight line and circle. (c) Mechanochromism of pattern scratching with a glass rod. (d) Original image.

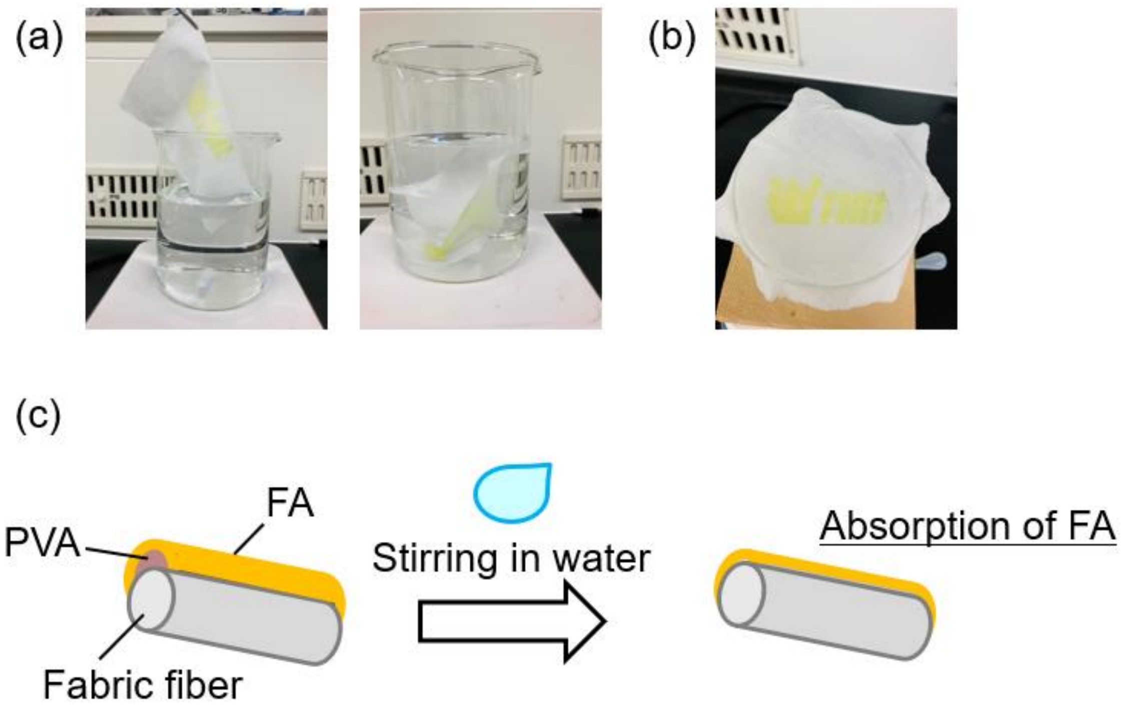

Figure S7. (a) Washing test for 15 min. (b) After washing. (c) Illustration of removal of PVA.
